# Supplementary material for: Resolving the full spectrum of human genome variation using Linked-Reads
Source: Genome Res. 2019 Apr;29(4):635–45. doi: 10.1101/gr.234443.118 (PMC6442396; doi:10.1101/gr.234443.118)
Supplement: Supplemental Material [file supp_gr.234443.118_Supplemental_longranger-2.2.zip › longranger-2.2.tar.bz2!/lr2.2-public/lib/stan/cmdstan/stan/doc/api/html/agrad_2rev_2functions_2square_8hpp_source.html]

Stan: src/stan/agrad/rev/functions/square.hpp Source File


|  |  |
| --- | --- |
|  | Stan  2.5.0  probability, sampling & optimization |


- Main Page
- Related Pages
- Namespaces
- Classes
- Files

- File List
- File Members

All Classes Namespaces Files Functions Variables Typedefs Enumerations Enumerator Friends Macros Pages

- src
- stan
- agrad
- rev
- functions

square.hpp

Go to the documentation of this file.

1 #ifndef STAN\_\_AGRAD\_\_REV\_\_FUNCTIONS\_\_SQUARE\_HPP

2 #define STAN\_\_AGRAD\_\_REV\_\_FUNCTIONS\_\_SQUARE\_HPP

3

4 #include <stan/agrad/rev/var.hpp>

5 #include <stan/agrad/rev/internal/v\_vari.hpp>

6

7 namespace stan {

8  namespace agrad {

9

10  namespace {

11  class square\_vari : public op\_v\_vari {

12  public:

13  square\_vari(vari\* avi) :

14  op\_v\_vari(avi->val\_ \* avi->val\_,avi) {

15  }

16  void chain() {

17  avi\_->adj\_ += adj\_ \* 2.0 \* avi\_->val\_;

18  }

19  };

20  }

21

47  inline var square(const var& x) {

48  return var(new square\_vari(x.vi\_));

49  }

50

51  }

52 }

53 #endif

stan::agrad::square

fvar< T > square(const fvar< T > &x)

**Definition:** square.hpp:15

stan::agrad::var::vi\_

vari \* vi\_

Pointer to the implementation of this variable.

**Definition:** var.hpp:40

v\_vari.hpp

var.hpp

stan::agrad::var

Independent (input) and dependent (output) variables for gradients.

**Definition:** var.hpp:27


---

[ Stan Home Page ]

*© 2011–2014,
Stan Development Team.*
